# Bio-Zirconium Metal–Organic Framework Regenerable Bio-Beads for the Effective Removal of Organophosphates from Polluted Water

**DOI:** 10.3390/polym13223869

**Published:** 2021-11-09

**Authors:** Kamal E. Diab, Eslam Salama, Hassan Shokry Hassan, Ahmed Abd El-moneim, Marwa F. Elkady

**Affiliations:** 1 Nanoscience Department, Institute of Basic and Applied Sciences, Egypt-Japan University of Science and Technology (E-JUST), New Borg El-Arab City, Alexandria 21934, Egypt; kamal.essam@ejust.edu.eg (K.E.D.); ahmed.abdelmoneim@ejust.edu.eg (A.A.E.-m.); 2 Department of Mechanical Engineering, University of Birmingham, Edgbaston, Birmingham B15 2TT, UK; 3 Environment and Natural Materials Research Institute (ENMRI), City of Scientific Research and Technological Applications (SRTA-City), New Borg El-Arab City, Alexandria 21934, Egypt; eslamsobhysalama@gmail.com; 4 Environmental Engineering Department, Egypt-Japan University of Science and Technology, New Borg El-Arab City, Alexandria 21934, Egypt; hassan.shokry@gmail.com; 5 Electronic Materials Researches Department, Advanced Technology and New Materials Researches Institute, City of Scientific Researches and Technological Applications (SRTA-City), New Borg El-Arab City, Alexandria 21934, Egypt; 6 Graphene Center of Excellence for Energy and Electronic Applications, Egypt-Japan University of Science and Technology (E-JUST) New Borg El-Arab City, Alexandria 21934, Egypt; 7 Chemical and Petrochemical Engineering Department, Egypt-Japan University of Science and Technology (E-JUST), New Borg El-Arab City, Alexandria 21934, Egypt; 8 Fabrication Technology Research Department, Advanced Technology and New Materials Research Institute, City of Scientific Research and Technological Applications (SRTA-City), New Borg El-Arab City, Alexandria 21934, Egypt

**Keywords:** zirconium metal−organic frameworks, MIP-202, diazinon, pesticides, Bio Zr-MOF beads, organophosphates wastewater

## Abstract

Organophosphate-based pesticides, such as diazinon, are among the most toxic organic contaminants to human and environment. Effective removal of diazinon from contaminated water sources is critical. Zirconium Metal−organic frameworks (Zr-MOFs) are promising candidates for the removal of organic contaminants from wastewater. Herein, we report the adequacy of a bio based Zr-MOF named MIP-202 for the removal of diazinon from water. On the other hand, the use of these materials in powder form is not workable, the development of scalable and economical processes and integrative of these materials onto beads is paramount for industrial processes. Hence, it was reported a scalable, bio aqueous solution-based preparation strategy for Bio Zr-MOF beads production. The composite material exposed identical reactivity under the same ambient parameters compared to powdered material in an aqueous solution. These results signify a critical procedure to an integrated strategy for organophosphates removal using bio-based MOFs, which demonstrates high potential for manufacturing applications such as continued removal of organophosphates from wastewater supplies.

## 1. Introduction

Agriculture and industrial manufacturing have contaminated freshwater sources with numerous inorganic and organic pollutants [1,2]. Finding ways to effectively alleviate this pollution is of paramount importance to protect and improve human, animal, environment, and aquatic life [3]. Organophosphate pesticides such as diazinon are one of the most famous organic contaminants in water supplies [4,5,6]. Diazinon is considered one of the most largely used pesticides due to its efficiency, cost-effectiveness [7]. It was found that it has a harmful impacts on human life and health which may cause damage to the nervous system, brain cancer, and respiratory paralysis [8]. Moreover, diazinon poisoning causes symptoms such as headaches, dizziness, tearing nausea, memory problems, and blurred vision [9]. As a result, the US Environmental Protection Agency (EPA), recognized the dangers of diazinon and has prohibited its products in the U.S. in 2004 [10]. However, the utilization of diazinon is ongoing in agriculture in many countries all over the world [5,11,12].

Many strategies have been explored for diazinon removal from wastewater including photocatalytic degradation and ozonation, coagulation, and chemical and biological oxidation [13,14]. However, each of these methods have drawbacks such as inefficient or moderate removal capacity, high operational energy costs, formation of toxic oxidation byproducts, and production of a large quantity of sludge [8]. Alternatively, adsorption is an efficient, comparatively inexpensive, and harmless technique that is a more convenient way of resolving diazinon removal [15,16]. Specifically, activated carbon materials have been inspected as sorbent material for diazinon removal [17,18]. Unfortunately, however, activated carbon is widely used for diazinon adsorption, and regeneration of activated carbon remains a challenge [19]. Furthermore, since activated carbon suffers from low volumetric capacity, the use of an excessive amount of sorbets is necessary, which results in a moderately expensive strategy [20,21]. In addition, activated carbon suffers from low diazinon adsorption capacity and often requires a long exposure time to reach the maximum adsorption capacity [22]. Therefore, recyclable, high capacity, and cost-effective materials for diazinon removal from wastewater must be intended to certify high efficient decontamination of water.

Recently, significant scientific attention was focused onto the hierarchically ordered mesoporous materials, as they are characterized by their high surface areas, uniform porosity, selectivity, storage capability, mass transport, and diffusion efficient rate [23]. It is expected that the chemical functionality characteristic and the hierarchical porous structure of both MOFs and COFs materials will afford both high uptake capacity and rapid uptake kinetics [23,24]. Covalent Organic Frameworks (COFs) are porous materials with high chemical stability and high specific surface area. COFs have been investigated for several applications such as optoelectronics, sensing, gas storage and separation, and water purification. Another category of crystalline materials is MOFs that are constructed from organic linkers and inorganic nodes, which have been utilized in numerous applications such as chemical separation, drug delivery, catalysis, gas storage, and separation [25,26,27,28,29]. The tailorable properties displayed by MOFs such as high internal surface area, and high water structural stability, ultrahigh porosity, also make them potential candidates for water purification [23,30]. MOFs have been investigated for inorganic and organic contaminant removal from water [30,31]. However, most of these reported MOFs are constructed from ligands extracted from petrochemical sources that are known for their intrinsic toxicity and availability [32,33]. Hence, there is a need to utilize bio-based MOFs prepared from available eco-friendly sources for water treatment to compromise the par excellence properties of MOFs and the biocompatibility.

Herein, we chose to investigate the efficiency of an amino acid-based MOF named MIP-202 as a bio-based zirconium MOF for the adsorption of diazinon from water. Notably, the MIP-202 is constructed from Zr-carboxylate bonds which have been highlighted for great interest due to their excellent stability [34]. In addition, MIP-202 is constructed from L-aspartic acid, cheap and available amino acid and could be prepared in scalable batches using ecofriendly methods [35]. MIP-202 was utilized for organic and inorganic contaminants removal and showed high efficiency in the powder form [36].

However, powdered forms of MOFs are typically poorly processable for practical applications; thus, formulated forms of MOFs are paramount. Many strategies have been developed to obtain processable forms of MOFs [37]. However, the reported studies usually have one or more of the following limitations, reduced surface areas when compared with the parent MOFs, resulting in low loading efficiency or not being biocompatible [38]. In addition, it is still challenging to prepare MOFs compounds that provide high surface area and fulfillment similar to the major MOFs using green strategies to get an industrial applicable form of MOFs powder [39]. For this, it was reported here a facile and scalable strategy for the synthesis of millimeter-sized bio-Zr MOF composite beads that are constructed from chitosan and sodium alginate in form of biocompatible beads, named chitosan–alginate beads (CA). Chitosan and alginate are considered one of the most widely used biomaterials [40]. Here, we used this method to formulate MIP-202 MOF powder in the form of processable beads. The adsorption performance of the fabricated MIP-202/CA beads has been investigated for diazinon remediation from waste solutions.

## 2. Materials and Methods

### 2.1. Materials

Thee starting chemical reactants Zirconium Chloride (Sigma Aldrich, 99.99%, St. Louis, MO, USA), Ethanol (Sigma Aldrich, <0.0005% water, St. Louis, MO, USA), l-Aspartic acid (Sigma Aldrich, ReagentPlus^®^, 99%, St. Louis, MO, USA), chitosan (Sigma Aldrich, St. Louis, MO, USA), sodium alginate (Sigma Aldrich, 99.99%, St. Louis, MO, USA), calcium chloride (Sigma Aldrich, St. Louis, MO, USA) and diazinon (Sigma Aldrich, analytical standard, St. Louis, MO, USA) have been used as it is.

### 2.2. Methods

#### 2.2.1. Synthesis of MIP-202 Powder

MIP-202 powder was synthesized using a modified green method to obtain colloidal stable nanoparticles. It is worth mentioning that MIP-202 is prepared without using any organic solvents that commonly used for MOFs synthesis. MIP-202 was synthesized by mixing ZrCl_4_ (1.15 g, 4.93 mmol) and l-aspartic acid (1.4 g, 10.52 mmol) and completely dissolving them in deionized water (10 mL). Then, the solution was transferred into a 100 mL round flask and refluxed at 90 °C under continuous stirring for 24 h. subsequently, the solution was kept out to cool down at room temperature and then washed several times with ethanol. Finally, the resulting precipitate was isolated and centrifuged to obtain the resulting precipitate. The white powder was collected and dried in a vacuum oven at room temperature for 24 h.

#### 2.2.2. Fabrication of MIP-202/CA Beads Composite

**Figure 1:**
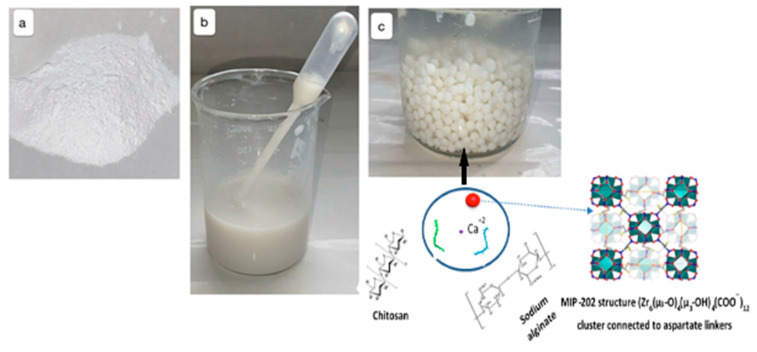
Fabrication of MIP-202/CA composite beads. (**a**) Powder sample of MIP-202. (**b**) MIP-202 powder sample with alginate solution. (**c**) White MIP-202/CA composite beads after washing.

One gram of MIP-202 was dispersed in a 100 mL of sodium alginate aqueous solution under stirring as indicated in Figure 1. Meanwhile, the curing solution was synthesized via dissolving 0.6 g of anhydrous CaCl_2_ in 100 mL of 0.1 mol/L chitosan aqueous solution. Then, the sodium alginate aqueous solution was dropped into the processing solution. After modelling (Figure 1), the collected synthetic beads were washed several times by distilled water. This followed by a freeze-drying step to produce the composite MIP-202/CA spherical beads. The chemical structures of the formulated beads MIP-202/CA composite is identified in Figure 1.

#### 2.2.3. Characterization Methods

Powder X-ray diffraction (PXRD) analysis were done using a D8 Bruker X-ray powder diffractometer, (CuKα1 radiation, λ = 1.54056 Å) at 40 kV and 40 mA and intensity data for 2θ from 20° to 70° over a period of 30 min. Fourier-transform infrared spectroscopy (FTIR) spectra were produced using Bruker Vertex 70 to explore chemical properties of MIP-202 after and before polymeric blend immobilization. Scanning electron microscopy (SEM) images were investigated using a Hitachi SU8030 FE-SEM (Dallas, TX, USA) microscope. The samples images were performed by transmission electron microscope (TEM) JEOL JEM-2100 200 kV (JEOL, Ltd. Akishima, Tokyo, Japan) by drop casting the MIP-202 powder and grounded MIP-202/CA composited beads and ethanol onto the 200-mesh copper TEM grid. The apparent surface areas were determined from nitrogen adsorption–desorption isotherms collected at 77 K by a Micromeritics Tristar II 3020. Thermal stability of the samples were performed using thermogravimetric analysis (TGA) as the samples were heated room temperature-700 °C with a rate of 10 °C/min under a constant flow of air. Freeze drying was performed with a critical point freeze dryer. Briefly, freeze drying was used in activation the water-soaked samples over a period of eight hours.

#### 2.2.4. Batch Adsorption of Diazinon from Polluted Water via MIP-202/CA Composite Beads

The diazinon adsorption efficiency of the MIP-202 bio-MOF/CA beads were investigated using batch technique. In batch method, 100 mg of different materials (MIP-202 bio-MOF, CA net beads, and MIP-202/CA composite beads) was mixed with 100 mL of 50 ppm diazinon solution concentration of pH = 7 at 22 °C for various time intervals (0–90 min) using a shaking incubator (Yellow line, Germany). The influence of processing parameters on the adsorption behavior of the MIP-202/CA beads including MIP-202/CA beads dosage (0.1–4.0 g/L), and initial pollutant concentration (10–100 ppm) were constituted. The accuracy of the data was proved by repeating all tests in triplicate and it was used the mean average values in the analysis processes.

### 2.3. Analytical Methods

After the diazinon adsorption processes, the adsorbent materials were separated using the centrifuge to control the final diazinon concentration via high-performance liquid chromatography (HPLC, Shimadzu, Japan). Subsequently, the supernatants were filtered using 0.25 µm syringe filters before injection into C18 column. The mobile phase was a mixture of methanol (88%) and ultrapure water (12%). The injection volume of diazinon solution was 20 µL. The flow rate and temperature of operation were 1 mL/min and 30 °C, respectively. Additionally, the detection wavelength of diazinon was 254 nm. Furthermore, the intermediate products of diazinon were set using liquid chromatography tandem mass spectroscopy (LC-MS/MS) analysis. A mixture of 65% methanol: 35% of 0.1% formic acid was utilized to detect the intermediates products of diazinon. The analysis experiments were done at flow rate and temperature of 0.4 mL/min and 35 °C, respectively.

The diazinon removal percentage via the adsorbent materials was determined from Equation (1) [40].

Removal% = ((*C_o_* − *C_e_*)/*C_o_*) × 100 (1)

where, *C_o_* is the initial diazinon concentration (mg/L); and *C_e_* is the diazinon concentration at equilibrium in aqueous solution (mg/L). The adsorption capacity (mg/g) of the diazinon was calculated by Equation (2) [40]:

*q_e_* = *V* (*C_o_* − *C_e_*)/m (2)

where, *V* is the diazinon solution volume (L); and *m* is the mass of adsorbent material (g).

#### 2.3.1. Equilibrium Isotherm Analysis for Diazinon Adsorption onto MIP 202/CA Composite Beads

Langmuir, Freundlich, and Temkin models were used to explain the performance of the adsorption processes of diazinon onto synthesized MIP-202 bio-MOF/CA beads. The Langmuir equation can be presented as following:

(3) $$\frac{C_{e}}{q_{e}}=\frac{1}{q_{m}K}+\frac{C_{e}}{q_{m}}$$

where, *q_e_* (mg/g) is the adsorbed amount of diazinon at equilibrium; *C_e_* (mg/L) is the concentration of the adsorbate at equilibrium; and *q_m_* (mg/g) and *K_L_* (L/mg) are Langmuir constants referred to the maximum monolayer adsorption capacity and adsorption energy, respectively. Moreover, the following Freundlich linear equation was applied to analyze the equilibrium data, by plotting log *q_e_* versus log *C_e_*.

Log *q_e_* = log *K_F_* + 1/*n_F_* log *C_e_* (4)

where *K_F_* and *n_F_* are Freundlich constants related to the adsorption capacity and intensity, respectively.

Temkin isotherm model was expressed for the adsorption behavior of diazinon onto the fabricated MIP-202 bio-MOF/CA beads as following [40].

*q_e_* = *B* ln *A* + *B* ln *C_e_* (5)

where, *A* (L/g) is the Temkin constant; and *B* = *RT*/*b* (J/mol) is constant referred to adsorption heat.

#### 2.3.2. Kinetic Models for Diazinon Adsorption onto MIP 202 Bio-MOF/CA Beads

To examine the diazinon adsorption technique on MIP-202 bio-MOF/CA composite beads from wastewater, the pseudo-first order, pseudo-second order, Elovich, and intraparticle diffusion kinetic models were utilized. The Lagergren first-order equation is represented as follows:

ln (*q_e_* − *q_t_*) = ln *q_e_* − *k*_1_*t* (6)

where, *q_e_* and *q_t_* (mg/g) are the amounts of adsorbed diazinon at equilibrium and at time *t* (min), respectively. *k*_1_ (min) expresses the constant rate of the first-order kinetic model. Moreover, the pseudo-second order kinetic model was used to analyze the adsorption kinetic data which can be expressed as following:

*t*/*q_t_* = (1/*k*_2_*q*^2^) + *t*/*q* (7)

where, *k*_2_ (g/mg·min) is the constant of the second-order rate. Furthermore, the following equation express Elovich model:

*q_t_* = *α* + *β* ln*t* (8)

where, *α* is (mg/g·min) the initial adsorption rate and *β* (g/mg) refers to the degree of the surface concealment and physical activation energy of adsorption. *α* and *β* can be obtained by calculating the slope and interrupt of the linear plot of *q_t_* against ln *t*, respectively. In a similar manner, the intraparticle diffusion affecting the diazinon adsorption processes from aqueous solution was expressed using Weber and Morris equation;

*q_t_* = *k_i_t*^1/2^ + *C* (9)

where, *k_i_* is the intraparticle diffusion rate constant. The value of *C* gives prediction about the boundary layer thickness. If intraparticle diffusion occurs, *q_t_* vs. *t*^0.5^ is linear and if the plot cross through the origin, the rate determination is only due to the intraparticle diffusion.

### 2.4. Reusability Study of the Fabricated MIP-202/CA Composite Beads

To evaluate the economic feasibility of the fabricated MIP-202 bio-MOF/CA beads in water treatment processes, the adsorption–desorption cycle was repeated for five times, where the adsorbent material was washed with distilled water and ethanol then dried in air for to be reused.

## 3. Results

### 3.1. Optimization and Characterizations of MIP-202 Nanopwders and MIP-202/CA Beads Composite

**Figure 2:**
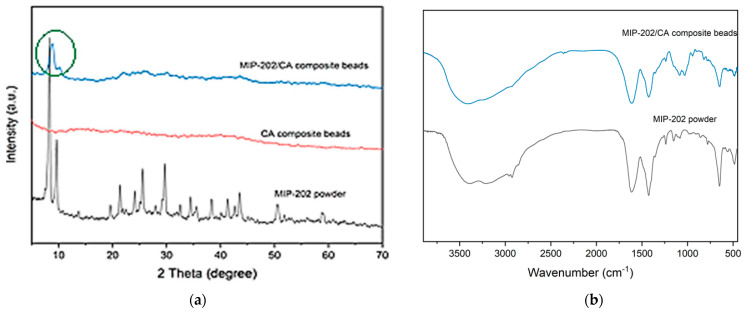
(**a**) Experimental PXRDs patterns of MIP-202 powder (black), chitosan-alginate composite beads (red), and MIP-202/CA composite beads (blue). (**b**) FTIR spectra of MIP-202 powder and MIP-202/CA composite beads.

MIP-202 MOF was selected as a bio-based zirconium MOF for the removal of diazinon from wastewater. MIP-202 is constructed from zirconium metal and aspartic acid through strong Zr(IV)−O bonds in which the 12-connected Zr_6_(μ_3_-O)4(μ_3_-OH)_4_ node and the L-aspartate ligand which explain extraordinary water stabilization due to Zr(IV)−O bonds [35]. So, MIP-202 has been investigated previously for the removal of dyes and heavy metals from the polluted water [36]. As, MIP-202 MOF material was produced using modified green technique, so its bulk phase purity was assured by the experimental PXRD as indicated from Figure 2a. It was evident from this figure that PXRD of the fabricated bio-MOF charactestricts with sharpness and high intensity peaks confirms its high crystallinity. The main distinguished peaks of MIP-202 were detected at 8.3°, 9.9°,14°, 20°, and 21.5°. These characterstics peaks identified the orientation planes of (111), (200), (222), (420), and (440), respectively. Meanwhile, the crystalline pattern of the free CA polymeric blended beads displayed weaker and broader peaks due to the amorphous state and fair crystallinity degree of the chitosan/alginate matrices. The spectra of the MIP-202/CA composite beads illustrated at Figure 2a that the addition of MIP-202 to the CA polymeric blend produces broader and reduced intensity peaks. This may be attributed to the inter or/and intra disturbance of crystal structure and hydrogen bonds of the CA in the synthesis processes of the MIP-202/CA composite bead. Accordingly, the proper incorporation of MIP-202 powder with CA polymeric blended beads at the fabricated MIP-202/CA composite beads was confirmed through both PXRD and FT-IR. comparing FT-IR spectra of both MIP-202 and its composite (Figure 2b), no noticeable difference between the pattern of pristine MIP-202 powder and MIP-202/CA composite that is confirmed by the proposed incorporation of MIP-202 particles with the polymeric blended matrix. This result is also confirmed from PXRD. The characteristic peaks of pristine MIP-202 powder are presented in the region of 600–1650 cm^−1^ in addition to the characteristic peaks of NH_2_ at 3380 cm^−1^ and 3495 cm^−1^ which decorate the internal pores of MIP-202 structure coming from the aspartic acid ligand. Accordingly, both PXRD and FTIR results showed the proper successful incorporation of MIP-202 powder with CA beads.

**Figure 3:**
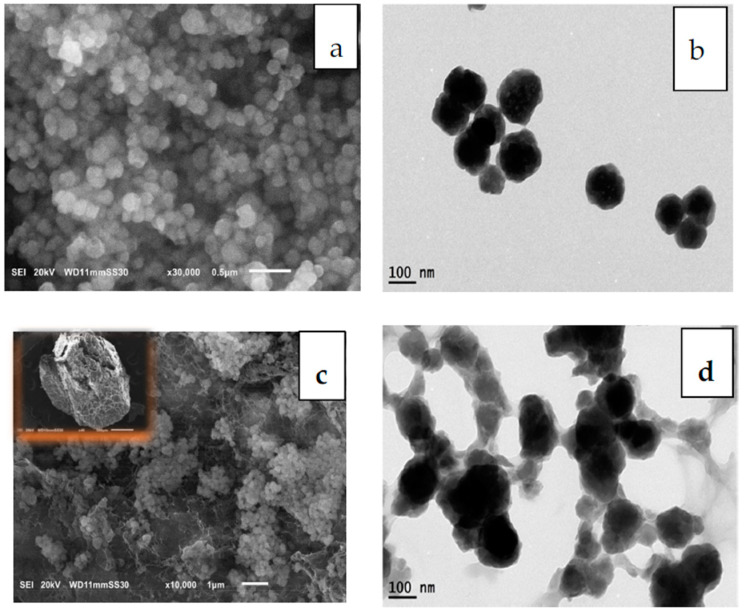
Morphological identification of pure MIP-202 nanoparticles and its composite. (**a**) SEM image of MIP-202 nanoparticles (**b**) TEM image of MIP-202 nanoparticles, (**c**) SEM image of MIP-202/CA composite bead. (**d**) TEM image of MIP-202/CA composite bead.

For the formation of MIP-202/CA composite beads, to better get a homogeneous solution of MIP-202 powder with the blended chitosan and alginate polymeric matrix, MIP-202 was prepared in nano size forms as confirmed from the SEM and TEM images (Figure 3a,b). SEM and TEM images of MIP-202/CA composite beads confirm homogeneous distribution of the MIP-202 MOF nano-powder resulting in a homogenous MOF composite bead (Figure 3). The TEM images showed the tight binding of the MOF nano-particles to the cross-linked chitosan alginate powder (Figure 3c,d).

**Figure 4:**
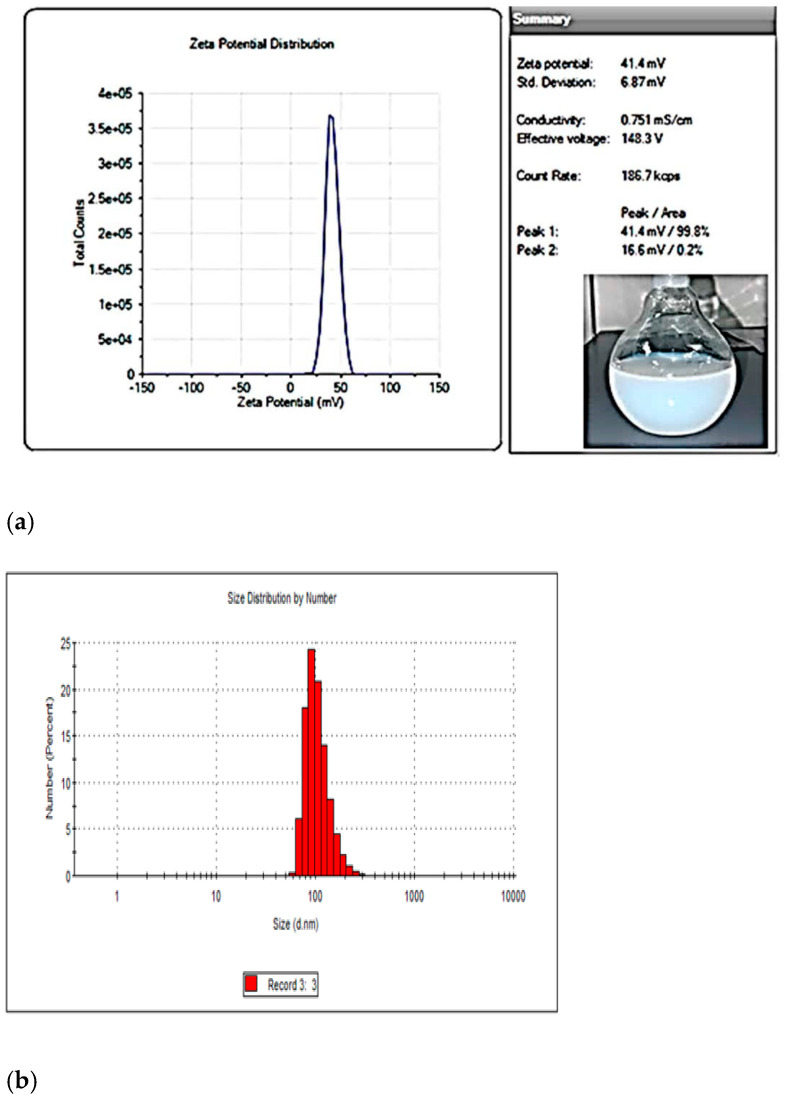
Colloidal stability of MIP-202 nanoparticles. (**a**) Zeta potential of MIP-202 nanoparticles, (**b**) Dynamic light scattering number analysis of MIP-202 nanoparticles.

In addition, the colloidal stability of the prepared MIP-202 particles was attained as shown from DLS measurements and zeta potential (Figure 4). The size of MIP-202 nanoparticles is proven by DLS and it shows a good agreement with the particle size measured from TEM images. It is interesting to note that the resulting MIP-202 nanoparticles powder showed high colloidal stability in water for several days. This is attributed to the high positive charge on MIP-202 nanoparticles measured using zeta potential with a value of 41.4 mv as shown in Figure 4. This high positive value of zeta potential allowed the nanoparticles of MIP-202 to be colloidally stable due to repulsion between particles in solution for several days as shown in Figure 1. The high colloidal stability of these nanoparticles prevents the sedimentation of MIP-202 particles while mixing the solution with alginate polymer solution which provided proper mixing, distribution, and incorporation of MIP-202 powder with alginate powder to provide an efficient mixed matrix of polymer and MIP-202 nanoparticles. The homogeneous distribution of MIP-202 nano-powder onto the CA polymeric blend was confirmed through imaging examination.

**Figure 5:**
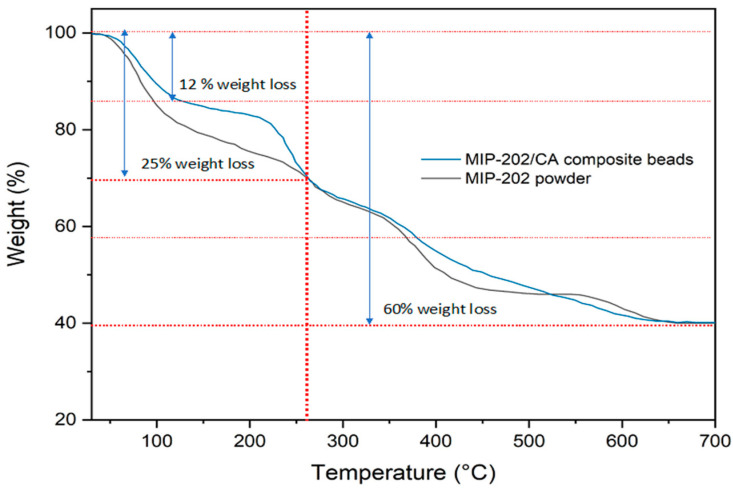
TGAs of MIP-202 powder (black), and MIP-202/CA composite beads (blue).

MIP-202 powder and MIP-202/CA composite beads was conducted using TGA (Figure 5). It is clear that both materials attain high thermal stability as both materials showed stability till 260 °C. For MIP-202 pristine powder, it shows first step thermal degradation of 12% weight loss at 100 °C, this is attributed to the release of adsorbed moisture and solvent adsorbed on the surface structure of the MOF. The same degradation step is observed in case of MIP-202/CA of weight loss around 12% but at 120 °C. The MIP-202/CA composite demonstrates second degradation step from 120 °C to 260 °C due to the release of water and residual solvent from the internal pores of MOF. The pristine MIP-202 shows also a second weight loss step from 100 °C to 260 °C due to the same reason. A gradual weight loss is observed for both pristine MIP-202 and MIP-202/CA after 260 °C to 700 °C due to the degradation of aspartic acid ligand and chitosan and alginate polymer in case of MIP-202/CA composite. A total weight loss of 60% is observed for both pristine MIP-202 and MIP-202/CA at 700 °C.

**Figure 6:**
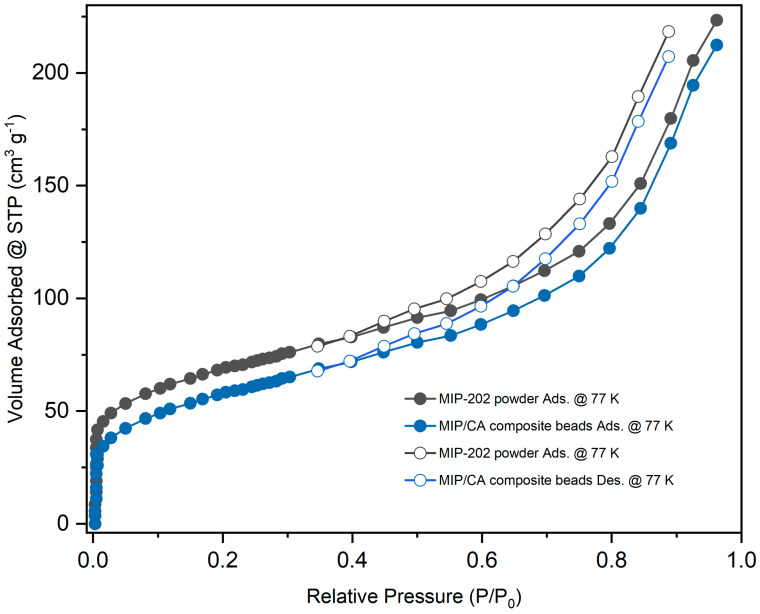
N_2_ adsorption−desorption isotherms of MIP-202 powder and its CA polymeric composite beads.

The nitrogen sorption isotherms of the resulting beads exhibited a detention of the porosity of MIP-202 embedded within the CA beads. The calculated BET surface areas was 180 m^2^/g for MIP-202 powder and 160 m^2^/g for MIP-202/CA composite beads, respectively, as evident from Figure 6. Moreover, the total pore volume and mean pore diameters were measured to be 0.061 cm^3^/g and 2.52 nm for the free MIP-202 powder, respectively. These values were estimated to be 0.029 cm^3^/g for the total pore volume and 12.24 nm for the average pore diameters of MIP-202/CA composite beads.

### 3.2. Diazinon Decontamination Using the Fabricated MIP-202/CA Composite Beads

The diazinon adsorption feasibility onto the fabricated MIP-202/CA composite beads from waste solutions onto the synthesized materials was performed using batch technique at room temperature.

#### 3.2.1. Influence of Contact Time on Diazinon Adsorption Processes

**Figure 7:**
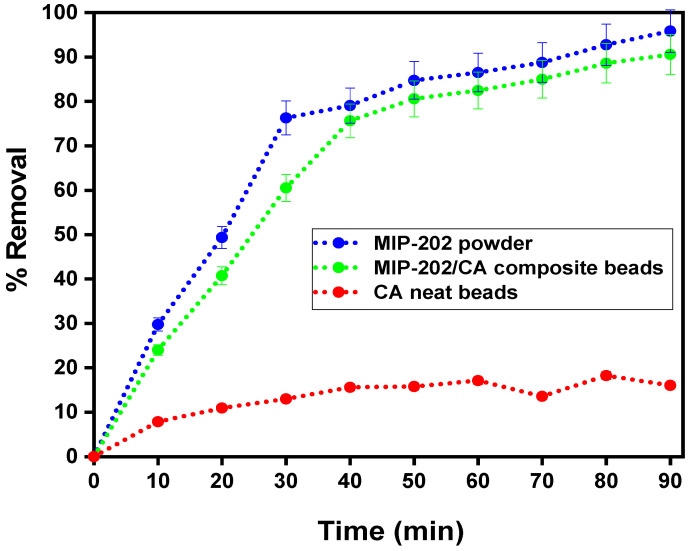
Effect of contact time on diazinon removal using MIP-202 powder, MIP-202/CA composite beads, and CA bio-beads at pH = 7, adsorbent dosage = 0.5 g/L, temperature = 22 °C, agitation speed = 300 rpm, and initial diazinon concentration of 50 mg/L.

Figure 7 demonstrates the influence of contact time of the diazinon adsorption percentage for the prepared MIP-202 bio-MOF powder, CA neat bio-beads, and MIP-202/CA composite beads at a time 0–90 min. It was observed that the neat CA bio-beads achieved the lowest adsorption performance of diazinon compared to MIP-202 powder and its composite beads. It is illustrated that, with increasing the contact time till the equilibrium state, the removal efficiency of diazinon onto the synthesized materials increased. The high diazinon adsorption rate in the first phase may be attributed to the functional groups and high surface area of the adsorbent materials which is binding with the diazinon molecule. The MIP-202/CA composite beads was recomended as an optimum adsorbent material for the supplementary experiments. The optimum contact time of the diazinon removal processes is recorded at 40 min, and the maximum diazinon adsorption percentage is 75.62%. At the second phase of adsorption, all active places of the adsorbent materials were saturated with diazinon molecules and non-significant adsorption results were achieved.

#### 3.2.2. Influence of MIP-202/CA Composite Beads Dosage on the Diazinon Adsorption Processes

**Figure 8:**
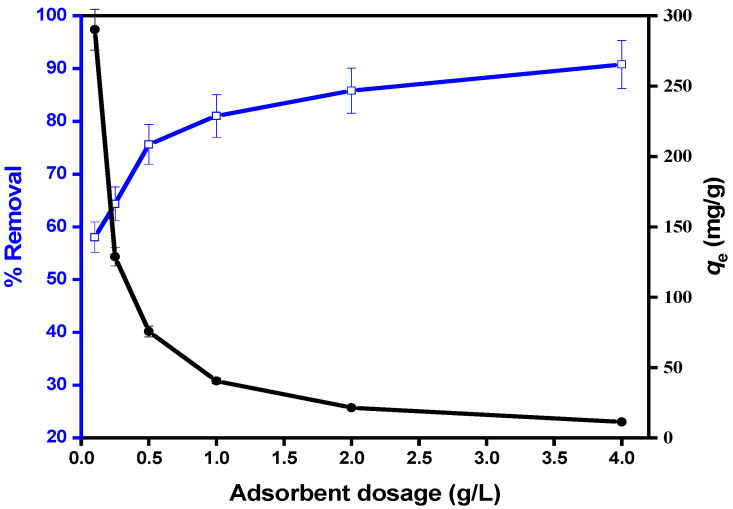
Effect of MIP-202/CA composite beads dosage on diazinon removal at pH = 7, contact time = 40 min, temperature = 22 °C, agitation speed = 300 rpm, and initial diazinon concentration of 50 mg/L.

One of the most significant factors in the adsorption processes for MIP-202/CA composite beads is the dosage due to its influences on the adsorbent beads’ capacity. The effect of the prepared MIP-202/CA beads dosage on the diazinon adsorption processes was evaluated after 40 min. Figure 8 illustrates that the diazinon adsorption process was improved as the amount of the synthesized MIP-202/CA beads increased from 0.10 to 4 g/L. conversely, the adsorption capacities of MIP-202/CA beads were decreased with increasing the dosage of adsorbent beads. This decline in the material adsorption capacity of diazinon at high beads dosage may be attributed to the unsaturated places of the MIP-202 bio-MOF/CA beads [36]. On the other hand, the dosage increase of MIP-202/CA beads improves the availability of more active sites for diazinon adsorption which enhances the removal ratio of diazinon. Thus, the optimum dosage of MIP-202 bio-MOF/CA beads was chosen as 0.5 g, which is determined as an economical dosage for the diazinon adsorption processes onto the fabricated beads [41].

#### 3.2.3. Influence of Initial Concentrations of Diazinon on the Adsorption Processes

**Figure 9:**
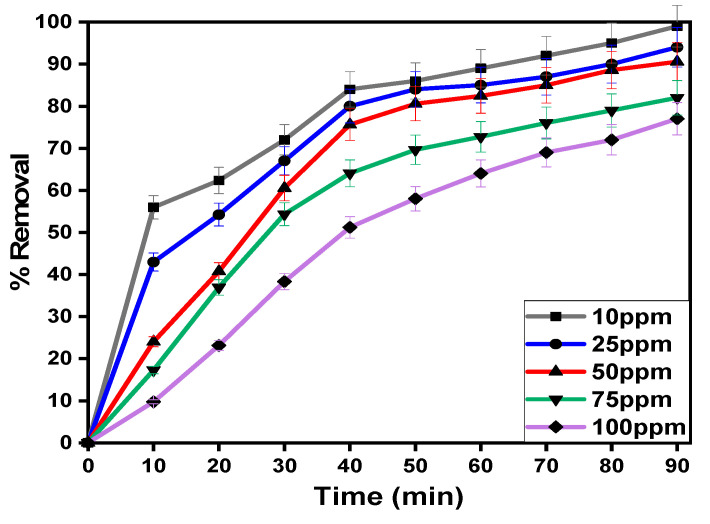
Effect of initial concentration of diazinon on removal process at pH = 7, contact time = 40 min, adsorbent dosage = 0.5 g/L, temperature = 22 °C, and agitation speed = 300 rpm.

The effect of diazinon initial concentrations was examined after 40 min with the initial concentration starting from 5 to 100 ppm in existence of 0.5 g as an optimum material dosage. In a similar manner, Figure 9 demonstrates that the diazinon adsorption capacities were enhanced with increasing the initial concentrations of diazinon from 10 to 100 ppm, which matched our reported studies [35,41]. This may be attributed to the saturation of the active regons of the MIP-202/CA composite beads increasing the initial diazinon concentrations. Accordingly, it was obvious that the MIP-202/CA beads is efficient as adsorbent agent for diazinon decontamination from waste solutions at different initial concentrations.

### 3.3. Equilibrium Isotherm Analysis for Diazinon Adsorption onto MIP 202/CA Composite Beads

**Figure 10:**
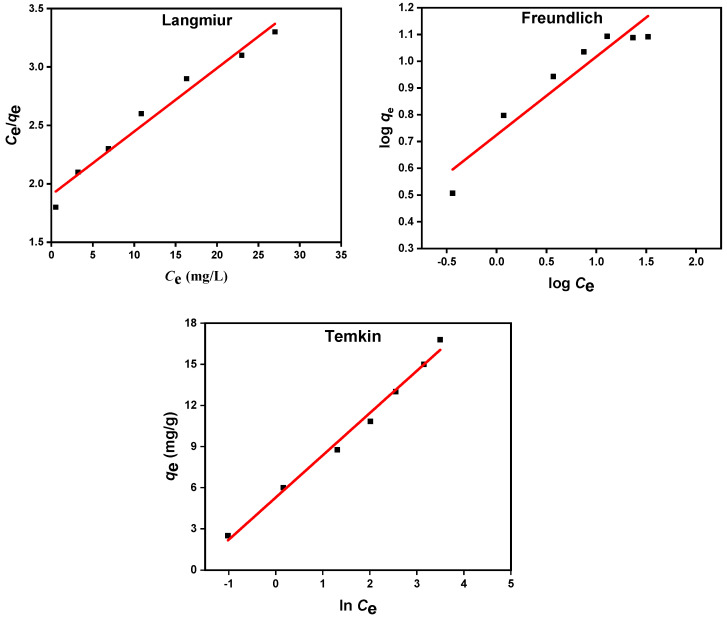
Isotherm models for diazinon adsorption onto MIP-202/CA composite beads.

The behaviors of adsorption processes of diazinon onto MIP-202/CA beads were investigated by Langmuir, Freundlich, and Temkin equilibrium models as indicated in Figure 10. Comparing the correlation coefficient of the three studied models at Table 1, it was demonstrated that Langmuir and Temkin models are the most fitted models for description the adsorption processes of diazinon onto the synthesized MIP-202/CA beads. In addition, the separation factor value (*R_L_*) in the range 0 to 1 illustrates the favorable adsorption processes of diazinon via Langmuir model [42]. However, the recorded Freundlich adsorption intensity value (*n_F_*) is 0.357, which is less than the unity, proving that the adsorption process of diazinon onto MIP-202/CA composite beads is unfavorable. On the other hand, the high value of Temkin correlation coefficient demonstrates that the equilibrium data of diazinon adsorption onto the synthesized composite bio-beads are fitted with Temkin isothermal model. Consequently, the Langmuir and Temkin models are the best favorable models for representing the monolayer chemical adsorption of diazinon on the fabricated MIP-202/CA composite beads.

#### Kinetic Models of Daizinon Adsorption onto MIP 202/CA Composite Beads

**Figure 11:**
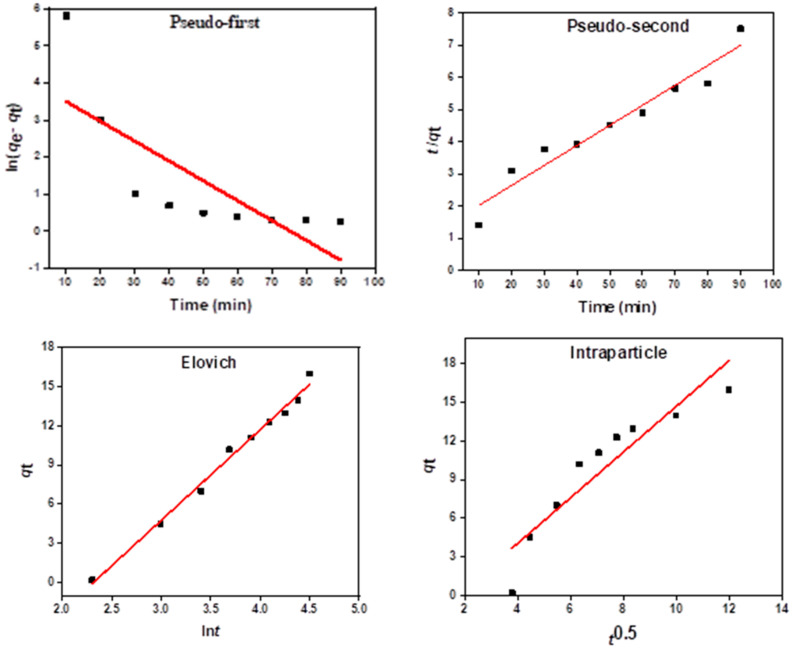
Kinetic models for diazinon adsorption onto MIP-202/CA composite beads.

To examine the diazinon adsorption mechanism onto the synthesized MIP-202/CA composite beads from waste solutions, the presented models of pseudo-first order, pseudo-second order, Elovich, and intraparticle diffusion kinetic models were achieved at Figure 11. Table 2 illustrated the comparable investigation of the *R*^2^ values for the linear plotting of the studied kinetic models for diazinon adsorption onto the synthesized MIP-202/CA beads from waste solutions. It is clear that the linearity of plotting *t*/*q_t_* versus time offers a high *R*^2^ for diazinon decontamination from wastewater indicates that the diazinon adsorption processes onto the fabricated MIP-202/CA beads agreed with the pseudo-second order kinetic model. Furthermore, the theoretical and experimental values of diazinon adsorption capacities onto the bio-beads are very close to each other. On the other hand, the correlation coefficient of Elovich equation recorded a high value of 0.921, which proposes that the diazinon adsorption process may be well explained using Elovich model. These data confirm that the studied diazinon removal process onto the fabricated MIP-202/CA beads is mainly controlled by chemisorption process [43].

### 3.4. Regeneration Studies of the Synthesized MIP-202/CA Beads

**Figure 12:**
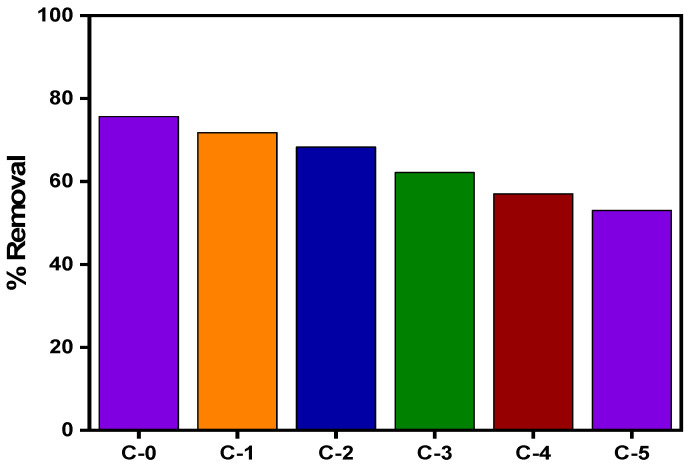
Reusability test of MIP-202/CA composite beads towards diazinon removal.

The study of regeneration the adsorbent material is one of the most important factors because it effects the total cost of the real applications [35]. Consequently, in order to investigate the regeneration process of the fabricated MIP-202/CA beads, it was reused five times for the diazinon adsorption in the batch experiments. The adsorption–desorption data confirm the ability of MIP-202/CA composite beads to be reused after five times, where the adsorption performance decreased to 54.12% as shown in Figure 12. This excellent adsorption behavior of MIP-202/CA composite beads may be due to the cooperative influence of MIP-202, chitosan, and alginate. In addition, the existence of excess hydroxide and amine groups on the surface of the beads leads to robust attraction between the fabricated MIP-202/CA composite beads and diazinon ions.

### 3.5. Comparison of Adsorption Capacity for MIP-202/CA Composite Beads with Other Adsorbent Materials

In this section, the capacity of mono-layer adsorption (*q_m_*) of diazinon onto the synthesized MIP-202/CA composite beads were compared with the diazinon adsorption capacities of different adsorbent materials as listed in Table 3. It was demonstrated from this table that the synthesized bio-beads have appropriate and promising efficiency for diazinon adsorption from waste solutions compared with the other reported adsorbent materials in the literature

## 4. Conclusions

In summary, we report for the first time the efficiency of a bio-based zirconium MOF named MIP-202 for the removal of diazinon from polluted water. Since the powder form of MOFs is not applicable in most industrial applications, an eco-friendly and scalable approach for fabrication of bio based composite beads from MIP-202 powder with chitosan and alginate biopolymers named MIP-202/CA beads was developed. The resulting, highly robust, and environmentally friendly MIP-202/CA composite beads demonstrated highly efficient removal of diazinon from polluted water. The maximum adsorption capacity was determined to be 17.77 mg/g and the adsorption behavior of diazinon was fitted with Langmuir model that indicated the monolayer adsorption onto the fabricated MIP-202/CA beads. The eco-friendliness, ease of synthesis, diazinon facile adsorption, and reusability of MIP-202/CA beads make this material a promising candidate to eliminate organophosphate from waste aqueous solutions. Moreover, we believe that our generalizable and scalable approach for integrating bio-MOFs onto bio-based polymers in form of processable bio-beads gives rise to a crucial step for widespread implementation of bio-MOFs material for water treatment industrial applications.

## Figures and Tables

**Table 1:** Isotherm parameters of Langmuir, Freundlich, and Temkin models for the diazinon adsorption onto the synthesized MIP-202/CA composite beads.

Langmuir Parameters			Freundlich Parameters			Temkin Parameters		
*q_m_* (mg/g)	*K_L_* (L/mg)	*R* ^2^	*K_F_* (mg/g)	*n* *_F_*	*R* ^2^	*A* (L/g)	*B* (J/mo)	*R* ^2^
17.776	0.030	0.973	0.533	0.357	0.793	0.133	32.626	0.993

**Table 2:** Pseudo-first order, pseudo-second order, Elovich and intraparticle diffusion kinetic models for diazinon removal onto MIP-202/CA beads.

Kinetic Model	Parameter	Value
**Pseudo-first order**	*q_exp_*.(mg/g)	80.71
	*q_theor_* (mg/g)	34.12
	*K*_1_ (min^−1^)	0.011
	*R* ^2^	0.743
**Pseudo-second order**	*q_exp_* (mg/g)	80.71
	*q_theor_* (mg/g)	75.62
	*K*_2_ (g/mg·min)	0.081
	*R* ^2^	0.935
**Elovich kinetic model**	*α* (mg/g·min)	−16.125
	*β* (g/mg)	9.221
	*R* ^2^	0.921
**Intraparticle diffusion kinetic model**	*C* (mg/g·min)	0.554
	*k_i_* (g/mg)	0.041
	*R* ^2^	0.843

**Table 3:** Comparison of mono-layer adsorption capacities of diazinon via different adsorbent nanomaterials.

Pollutant	Adsorbent Material	Optimized Conditions	Adsorption Capacity (mg/g)	Reference
**Diazinon**	MIP-202/CA beads	Dosage = 0.5 g/L Conc. = 50 mg/L Time = 40 min	17.77	Present work
	Magnetic multi-walled carbon nanotubes	Dosage = 0.1 g/L Conc. = 8 mg/L Time = 30 min	3.89	[43]
	Activated bentonite	Dosage = 0.5 g/L Conc. = 10 mg/L Time = 120 min	5.56	[44]
	Phosphoric acid coconut shell biochar	Dosage = 1 g/L Conc. = 10 mg/L Time = 120 min	10.33	[45]
	Nano-TiO_2_ and polypropylene	Dosage = 2.25 g/L Conc. = 10 mg/L Time = 32.2 min	1.24	[46]
	Magnetic guar gum	Dosage = 1 g/L Conc. = 5 mg/L Time = 60 min	7.03	[47]
	Magnetic guar gum-montmorillonite	Dosage = 1 g/L Conc. = 5 mg/L Time = 60 min	9.73	[48]
	Acid treated zeolite	Dosage = 3 g/L Conc. = 50 mg/L Time = 20 min	15.10	[49]
	Modified zeolite by Cu2O	Dosage = 2 g/L Conc. = 50 mg/L Time = 20 min	61.73	[49]
	NH_4_Cl-induced activated carbon	Dosage = 0.3 g/L Conc. = 20 mg/L Time = 30 min	25.00	[50]
	Walnut shell-modified activated carbon	Dosage = 1 g/L Conc. = 40 mg/L Time = 60 min	34.31	[51]
	Fe-TiO_2_/Bent-Fe	Dosage = 0.5 g/L Conc. = 16.74 mg/L Time = 30 min	27.03	[52]

## Data Availability

Not applicable.
